# Evaluation of Outcomes After Early Versus Late Initiation of Continuous Renal Replacement Therapy in Adult Patients With Sepsis-Associated Acute Kidney Injury

**DOI:** 10.7759/cureus.111819

**Published:** 2026-06-30

**Authors:** Soumya Sankar Nath, Hina Ashraf, Rachana Gupta, Sharif Alam, Virendra Kumar

**Affiliations:** 1 Anesthesiology and Critical Care Medicine, Dr. Ram Manohar Lohia Institute of Medical Sciences, Lucknow, IND

**Keywords:** anion-gap metabolic acidosis, continuous renal replacement therapy (crrt), kdigo criteria, length of hospital stay, length of stay in icu, sepsis-associated acute kidney injury, severe uremia

## Abstract

Background and aim: Prospective studies comparing outcomes following early versus late initiation of continuous renal replacement therapy (CRRT), labeled as per Kidney Disease: Improving Global Outcomes (KDIGO) guidelines, in patients with sepsis-associated acute kidney injury (S-AKI) are lacking. The aim was to compare the 28-day mortality rate, renal recovery rate, and length of stay in ICU and hospital among patients of S-AKI with early versus late institution of CRRT.

Patients and methods: Sixty consenting adult patients (18-65 years) of S-AKI needing CRRT were screened. In the early CRRT group, it was instituted in KDIGO stage I AKI with complications (either raised serum potassium, metabolic acidosis, uremia, or acute pulmonary edema) or stage II. The late CRRT group was for patients with KDIGO stage III or stage II who could not have CRRT started within two hours.

Results: The data from 32 patients were collected and analyzed in the study. Demographic parameters and illness severity were comparable between the early and late CRRT groups. The length of ICU stay, need for blood transfusion, renal recovery, and the requirement for RRT on day 28 were comparable between the two groups. The 28-day mortality rate in the early CRRT group was lower than that in the late CRRT group (4/25% versus 7/43.75%, p-value 0.048).

Conclusion: Early initiation of CRRT (KDIGO stage I/II) in patients with S-AKI leads to lower 28-day mortality compared to those in whom CRRT was initiated at a late stage (III).

## Introduction

Sepsis-associated acute kidney injury (S-AKI) has been characterized as AKI in patients with sepsis and in the absence of substantial contributing factors supporting the diagnosis of AKI or distinguished by the coexistence of both Sepsis-3 and Kidney Disease: Improving Global Outcomes (KDIGO) criteria [1,2]. The pathophysiology of sepsis is complex and distinct. Similarly, the pathophysiology of S-AKI differs from that of other AKI phenotypes. Moreover, though sepsis is the most frequent cause of AKI, any cause of AKI also makes the patient highly susceptible to sepsis [3]. S-AKI is a frequently encountered complication among patients with sepsis, with mortality varying from 15-60% [4].

S-AKI is thought to result from inflammatory damage-induced perturbations in microcirculatory oxygen delivery, possibly due to decreased flow and restricted diffusion in the presence of organ edema and inflammation. Although the exact implications of these disturbances in microcirculation are yet to be fully understood, it is evident that sepsis intensifies the expression of inflammatory cytokines and promotes leukocyte activity. These together lead to capillary plugging and the formation of microthrombi. This causes the production of reactive oxygen species (ROS) and the induction of nitric oxide synthase. ROS and nitric oxide damage the endothelial barrier and the glycocalyx, causing structural and functional changes in the kidneys [5]. Moreover, during sepsis, the Toll-like receptors identify the pathogen-associated molecular pattern and damage-associated molecular pattern, which elicit tissue inflammation followed by injury. These, in turn, provoke thrombosis of the microvasculature. There is increased vascular permeability and interstitial edema, which, in turn, damages renal microcirculation, leading to venous congestion of the kidneys and, thus, a reduction in glomerular filtration [6]. Further, it was reported that in patients with septic shock, the concentrations of circulating inflammatory cytokines correlate with mortality. Sood et al. reported that AKI, which could be treated early, led to enhanced survival in sepsis [7]. Therefore, measures to reduce volume overload, which will avoid the resultant renal venous congestion and interstitial edema, along with adequate removal of inflammatory mediators from the circulation, might prove life-saving in a condition with otherwise poor outcomes. In critical care, CRRT is currently preferred over intermittent renal replacement therapy (RRT) because it maintains stable hemodynamic parameters, provides efficient volume control with fluid and solute clearance, corrects acid-base and electrolyte disorders, and removes pro-inflammatory mediators [8].

Both early and late initiation of continuous RRT (CRRT) have their pros and cons. Early institution of CRRT ensures early metabolic and uremic control and prevents fluid overload. Preventing fluid overload and acidemia protects the kidneys and other organs from injury. It is claimed that early initiation of CRRT can stabilize the internal milieu and eliminate inflammatory mediators [9]. Hence, early CRRT may control excessive inflammation, thus decreasing kidney injury and, possibly, improving patient survival. On the other hand, delayed initiation might spare some patients the potential ill effects of CRRT, whose kidneys had a high likelihood of recovery without it. In critically ill patients with AKI, the STARRT-AKI (Standard versus Accelerated Initiation of Renal-Replacement Therapy in Acute Kidney Injury) trial failed to find a lower risk of death at 90 days in those who received accelerated RRT compared to those who received RRT as per the standard-strategy group. The patients were of mixed diseases and not specifically of SA-AKI [10]. As of now, prospective studies comparing outcomes following early versus late initiation of CRRT, as per KDIGO guidelines, in patients with S-AKI are lacking [11]. So, the primary objective of the present study was to compare the 28-day mortality after initiation of CRRT among adult patients with S-AKI who received early versus late initiation of CRRT. The secondary objectives were to compare renal recovery rates and lengths of stay in the intensive care unit (ICU) and hospital among critically ill patients with S-AKI who initiated CRRT early or late.

## Materials and methods

This study is designed as a hypothesis-generating, phenotype-focused, single-center, prospective, observational, open-label study and was performed in adult patients of S-AKI who received CRRT in the ICU of a teaching hospital (Dr. Ram Manohar Lohia Institute of Medical Sciences, Lucknow, India) during the study period of 18 months after getting approval from the Institute Ethical Committee (IEC No. 57/18, dated 09/04/19) and registering in the Clinical Trials Registry-India (CTRI Registration No.-CTRI/2024/07/071659). Informed consent was obtained from the nearest relative of each patient before enrolling them in the study. Adult patients (18-65 years) with S-AKI, as per KDIGO classification, and whose nearest kin agreed to CRRT, were recruited for the study. Those with pre-existing chronic kidney disease, previous renal replacement therapy, do-not-resuscitate (DNR) status, CRRT instituted for indications other than KDIGO guidelines-designated AKI, CRRT stopped within 24 hours, death before completion of CRRT, and post-renal transplant patients were excluded from the study.

**Figure 1:**
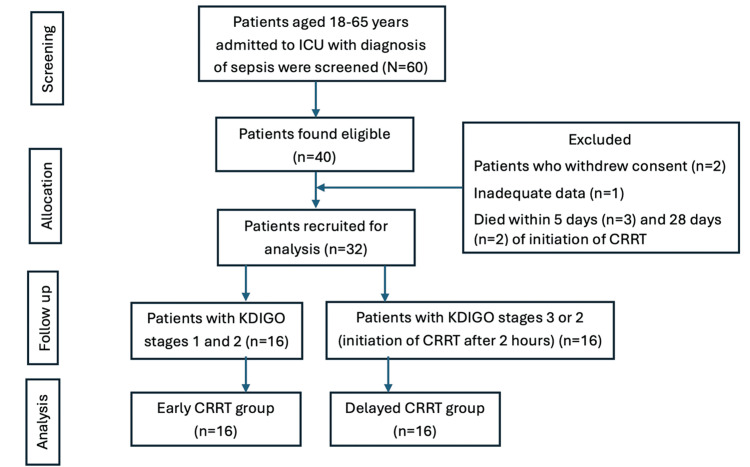
Flowchart of the study protocol KDIGO: Kidney Disease: Improving Global Outcomes; CRRT: continuous renal replacement therapy; DNR: do-not-resuscitate

We observed two groups of 20 patients, each according to the KDIGO stage in which CRRT was initiated. The two groups of patients were labeled as early or late CRRT. The early CRRT group was when CRRT was instituted in patients with SA-AKI and KDIGO stage I AKI with hyperkalemia (serum potassium > 5.5 mEq/L), metabolic acidosis (pH < 7.2), features of uremia (encephalopathy), acute pulmonary edema, or KDIGO stage II. A maximum of two hours was allowed between the clinical decision to initiate CRRT and its actual commencement. The late CRRT group was when CRRT was initiated in patients of SA-AKI in KDIGO stage III and stage II, if CRRT could not be started within two hours. Figure 1 shows the flowchart of the study protocol.

Sample size calculation

Based on data from a previous study, the sample size was estimated with a 95% confidence interval, a power of 80%, and an assumed non-response rate of 10% [12]. Thus, the calculated sample size was 16 patients in each group. To compensate for dropouts, it was decided to recruit 20 patients per group.

Upon admission to the ICU at our institution, all patients underwent a thorough physical examination and laboratory investigations. Arterial blood gas analysis was done at the time of admission into the ICU and repeated twice a day during the ICU stay, or more frequently as deemed by the on-duty doctor. Disease severity was evaluated using the Acute Physiology and Chronic Health Evaluation II (APACHE II) score [13]. CRRT was initiated by the intensive care team per existing ICU protocols. Before commencing CRRT, different blood and urine variables were documented. The treating ICU team decided on a CRRT prescription. The CRRT effluent dose was noted once daily during ongoing CRRT therapy. CRRT can be stopped if renal recovery, defined by urine output (UO), meets the stipulated criteria (UO > 400 mL in 24 hours without diuretics or UO > 2100 mL in 24 hours with diuretics). If the criteria for terminating CRRT were not met, CRRT was continued for at least five days. Citrate was used for regional anticoagulation. The filter lifespan, length of ICU stays, need for transfusion, renal function recovery, duration of CRRT, and other parameters were assessed for every patient undergoing CRRT in the ICU. Serum creatinine (Sr Cr), RRT requirement, and mortality at the end of 28 days were recorded.

A hemodialysis catheter was inserted through the femoral or right internal jugular vein. Continuous venovenous hemodiafiltration was done using the Gambro Prismaflex (Baxter, Sweden) machine. CRRT commenced with 100 ml/min of blood flow, progressively increasing to 150 ml/min. The ultrafiltration dose was set at 40 ml/kg/h. In addition, the CRRT circuit was changed when the blood pump was stopped or when the transmembrane pressure exceeded 300 mmHg. The patients were weighed on an in-bed scale every morning, and the ultrafiltration dose was adjusted accordingly.

We used CRRT-specific commercial solutions (Regiocit, Baxter India Private Limited) for replacement, as they are recommended over custom-made solutions due to the risks of compounding errors and a break in sterility. The fluid used in the regional citrate anticoagulant is Regiocit™ (Baxter, Sweden) (sodium chloride and sodium citrate) as a pre-blood pump. Prismasol™ (Baxter, Sweden) was used as a post-blood pump. Biphozyl™ (Baxter, Sweden) fluid was used as a dialysate. The pre-pump fluid was given at 1000ml/hr, and the post-pump fluid was given at 300ml/hr. Calcium gluconate infusion was started and titrated to maintain serum ionic calcium between 1 and 1.2 mmol/L.

The following data were collected: demographics (gender, age, and admission dates); admission status; 28-day outcome; CRRT-related (time of onset, duration, vascular access, and mode of anticoagulation); and baseline values (collected within 24 hours of admission) like blood urea and Sr Cr, APACHE II scores, C-reactive protein (CRP), and serum bicarbonate (HCO₃⁻) [13]. Further, UO per 24 hours and KDIGO staging were noted. Renal recovery, or the absence of it, was based on the levels of Sr Cr and blood urea at discharge or the last report before demise. We used 28-day death as the primary endpoint and 28-day renal recovery as a secondary endpoint.

It was deemed that kidney function had recovered when the Sr Cr level or creatinine clearance (CrCl) began to fall, calculated from timed UO over six hours when urine flow exceeded 30 ml per hour. CrCl was intermittently monitored to determine whether further continuation was warranted. CRRT was continued if the CrCl was less than 12 ml/min and stopped if it exceeded 20 ml/min. For intermediate values of CrCl, the decision to continue or discontinue was left to the clinician. Further, the treating physicians were free to manage RRT in patients with kidney failure persisting beyond 28 days after recruitment or in those who were shifted out of the ICU before day 28, as decided by the treating physicians, because of an otherwise improved condition [14].

Statistical analysis

Continuous and categorical variables were expressed as mean ± standard deviation (SD) and as numbers and percentages, respectively. The Student t-test and the χ2 test were used to compare the continuous and categorical variables of baseline characteristics of the two groups. IBM SPSS Statistics for Windows, Version 26 (Released 2018; IBM Corp., Armonk, New York, United States) was used for the statistical analysis.

## Results

Sixty patients were screened for the study; 40 met the criteria for CRRT and were recruited. Out of 40 patients, three patients died within five days of CRRT initiation, two patients died before 28 days, one patient had inadequate data collection, and two patients' kin withdrew their consent before completion of the study. Hence, data from 32 patients were collected and analyzed in the study.

Table 1 compares the demographic and baseline parameters between the two groups. Demographic parameters and illness severity, as measured by APACHE II scores, were comparable between the early and delayed CRRT groups. The pre-CRRT mean Sr Cr was significantly higher, and UO was significantly lower in the late CRRT group compared to the early CRRT group. The baseline Sr Cr was lower, and UO before the start of CRRT was higher in the early CRRT group compared to the late CRRT group. Because the KDIGO AKI staging criteria depend on S Cr and UO, it is clear that these two parameters were significantly different between the two groups.

**Table 1:** Comparison of demographic and baseline parameters between the two groups APACHE: Acute Physiology and Chronic Health Evaluation; CRRT: continuous renal replacement therapy *p < 0.05: statistically significant

Parameter	Group	Mean±SD	P-value
Age (years)	Early	57.06± 21.16	0.285
	Late	54.71±15.60	
Predicted body weight (kg)	Early	62.06±4.61	5.14
	Late	60.81±5.14	
Gender (number of males)	Early	9	0.51
	Late	6	
APACHE II score	Early	25.50±4.97	0.697
	Late	23.18±4.30	
Duration of CRRT (days)	Early	5.00±1.71	0.355
	Late	4.53±1.46	
Effluent dose (ml/kg/hr)	Early	25.63±4.57	0.345
	Late	26.41±5.44	
Baseline serum creatinine (mg/dL)	Early	1.05±0.42	<0.001*
	Late	2.99±2.97	
Urine output before CRRT (ml)	Early	301.88±62.96	<0.001*
	Late	201.94±146.23	

Table 2 shows the comparison of various parameters like blood urea nitrogen (BUN), serum CRP, Sr Cr, HCO_3_, and UO between the two groups on different days from day 1 to day 5 of CRRT. We found that on day 2, Sr Cr and bicarbonate were significantly lower in the early CRRT group compared to the delayed CRRT group. All other factors were comparable in the two groups from day 1 to day 5. Thus, HCO_3 _and creatinine correction occurred more rapidly in the early CRRT group than in the delayed CRRT group. 

**Table 2:** Comparison of various parameters between the two groups on day 1 to day 5 of CRRT CRRT: continuous renal replacement therapy; BUN: blood urea nitrogen; CRP: C-reactive protein; HCO_3_: serum bicarbonate *p < 0.05: statistically significant

Day of CRRT	Groups	Parameters									
		BUN (mg/dl) Mean±SD	P-value	Sr creatinine (mg/dl) Mean±SD	P-value	CRP (mg/L) Mean±SD	P-value	HCO_3_^-^ (mEq/L) Mean±SD	P-value	Urine output (ml) Mean±SD	P-value
Day 1	Early	63.69±22.74	0.56	3.95±1.46	0.52	60.44±24.23	0.37	19.41±2.56	0.70	355.44±192.42	0.13
	Late	69.59±20.85		5.01±1.98		51.24±26.92		19.62±2.62		352.06±250.88	
Day 2	Early	58.77±18.75	0.98	4.06±1.32	0.006*	55.88±23.96	0.94	20.83±2.14	<0.001*	547.19±262.38	0.55
	Late	60.45±17.76		4.34±1.08		52.94±22.78		20.47±4.61		351.25±300.31	
Day 3	Early	66.63±23.88	0.89	4.05±1.20	0.31	56.94±25.54	0.43	18.58±2.99	0.06	818.31±382.57	0.68
	Late	55.54±25.48		3.59±1.01		47.59±22.75		21.17±4.51		451.65±445.79	
Day 4	Early	53.0±19.7	0.34	4.11±1.28	0.89	54.25±29.41	0.63	19.48±2.52	0.98	971.88±531.56	0.80
	Late	54.35±25.02		4.36±1.15		48.47±31.42		19.31±2.65		710.47±562.65	
Day 5	Early	48.06± 26.21	0.61	4.18±1.52	0.41	59.44±27.61	0.94	20.62±2.72	0.688	1027.69±621.46	0.55
	Late	58.76± 23.65		3.98±1.26		53.48±27.32		19.59±2.8		735.0±586.77	

Table 3 compares various outcome parameters between the two study groups. The length of ICU stay, need for blood transfusion, renal recovery at day 28, and the requirement for RRT at day 28 were comparable between the two groups. We found lower 28-day mortality in the early CRRT group compared with the late CRRT group (4/25% versus 7/43.75%, p value 0.048). The relative risk and the odds ratio for mortality between groups were 0.57 (95%CI 0.21-1.58) and 0.43(95% CI 0.095-1.925), respectively.

**Table 3:** Comparison of various outcome parameters between the early and late CRRT groups CRRT: continuous renal replacement therapy *p < 0.05: statistically significant

Parameter	Group	Mean±SD	P-value
Length of ICU stay (Days)	Early	13.81±3.78	0.980
	Late	13.76±3.80	
Transfusion requirement	Early	1.56±0.51	1.00
	Late	1.53±0.51	
Renal recovery at 28th day	Early	8(50%)	0.508
	Late	6(37.5%)	
RRT requirement at day 28	Early	10(62.5%)	1.00
	Late	10(62.5%)	
Mortality at day 28	Early	4(25%)	0.048*
	Late	7(43.75%)	

## Discussion

We observed that the demographic parameters and APACHE II scores, which assessed the severity of illness among the patients, were comparable between the early and late CRRT groups (25.5 ± 4.97 and 23.18 ± 4.3, respectively; p=0.69) (Table 1). Renal recovery at day 28 (50% in the early CRRT group versus 37.5% in the delayed CRRT group, p=0.325) (Table 1) and the requirement for RRT at day 28 (62.5% in both groups, p=1.0) were comparable between the two groups (Table 3). We found that there was a lower 28-day mortality among patients in the early CRRT group (25%) compared to the delayed CRRT group (43.75%) (p=0.048) (Table 3).

Although there are several studies and systematic reviews that compared the outcomes, like mortality and renal recovery, among patients of AKI who underwent early or late CRRT, most of them are either retrospective, with arbitrary definitions of early/late initiation of CRRT, or involve patients other than those afflicted with SA-AKI. Thus, ours is the first prospective study to evaluate renal recovery and mortality on day 28, specifically among patients with SA-AKI who underwent early/late initiation of CRRT.

The observations of our study are similar to those of Wang X et al. (2012) and Vats HE et al. (2011) [15,16]. Wang X et al. (2012) described in a meta-analysis of 15 studies that among those who underwent CRRT, the early CRRT group had significantly lower mortality compared with delayed CRRT (27.8% versus 43.0%) [15]. The characterization of early and delayed commencement of CRRT varied in different reports included in the meta-analysis. For instance, early CRRT was labeled when RRT was started within 12 hours if UO was < 30 ml/kg/h, whereas late CRRT was labeled when RRT was started when urea > 40 meq/L or serum potassium > 6.5 mmol/L [17]. RIFLE criteria (risk) were labeled early. In contrast, it was late when RRT was initiated in the failure class [18]. Further, early CRRT was initiated when blood urea was below 21.4 mmol/L, and the late group was when blood urea exceeded 21.4 mmol/L, and so on [19]. Because the early and delayed CRRT groups were labeled differently across the studies included in the meta-analysis, the meta-analysis's conclusions are uncertain. None of the studies included in the concerned meta-analysis defined ‘early’ and 'late' RRT based on KDIGO grades for renal failure, as the KDIGO guidelines were published after this meta-analysis [14]. Further, the population studies included mixed ICU patients. The authors themselves agreed that, though they found that early CRRT had a beneficial effect on ICU mortality, the study suffered from publication bias, as they could include only a handful of studies; of the 15 trials, only three were randomized controlled trials, and the rest were retrospective studies [15].

In the retrospective study by Vats HE et al. (2011), the diagnosis of AKI warranting CRRT did not meet KDIGO guidelines (because KDIGO guidelines were published in 2014). They labelled AKI as a Sr Cr level ≥ 2.0 mg/dl when the baseline value was < 1.5 mg/dl, or a Sr Cr level ≥ 2.5 mg/dl if the baseline value was > 1.5 mg/dl. It included a mixed ICU population and not specifically patients of SA-AKI. They further defined early CRRT as those started within 6 days of AKI confirmation, whereas the delayed CRRT group was those started beyond six days of AKI diagnosis. They reported that the risk of the patient dying in the delayed CRRT group was greater than in the early CRRT group, with the former having an odds ratio of 11.66 (95% CI of 1.26-107.91, p-value 0.03) [16].

We chose KDIGO stages at which CRRT was instituted to distinguish the early CRRT group from the delayed CRRT group [2]. KDIGO stages had better sensitivity for labeling AKI and robust capability to envisage the outcome, so it is sensible to utilize KDIGO stages at which the RRT was initiated for labeling early/late CRRT groups.

In the ELAIN (Early vs Late Initiation of Renal Replacement Therapy in Critically Ill Patients With Acute Kidney Injury) study, the authors recruited patients with septic shock, intractable fluid overload, or acute respiratory distress syndrome (ARDS), or a sepsis-related organ failure assessment (SOFA) score of two or more with KDIGO stage II AKI. They randomized them into two groups: early (RRT began within eight hours of confirmation of stage II AKI) and late (RRT began within 12 hours of confirmation of stage III AKI). They found that when CRRT was started early, 90-day mortality was significantly lower than in the late group (39.3 vs. 54.7%, p-value=0.03). Further, early initiation of CRRT led to the recovery of renal functions at day 90 (53.6% vs. 38.7%, p < 0.02), reduced mean length of mechanical ventilation (125.5 hours vs. 181 hours, p < 0.02), and decreased duration of stay (51 days vs. 82 days, p < 0.001). However, the requirement for RRT at day 90 (13.4% vs. 15.1%) and the total days spent in ICU (19 vs. 22) were comparable [20]. It is worth mentioning that most of the patients recruited in the study were surgical patients, unlike our cohort of SA-AKI patients.

Wu X et al. conducted a retrospective study of SA-AKI patients undergoing early (KDIGO stage I/II) and late CRRT (KDIGO stage III). They found that, although the CRRT duration was shorter in the early CRRT group, the lengths of ICU and hospital stays, 28-day and 90-day mortalities, and 28-day and 90-day RRT disengagement or dependent rates were comparable across groups. Even the 90-day cumulative survival rates were comparable [21].

Fan Y et al. (2022) performed a retrospective study where the results of different approaches of RRT, RRT versus non-RRT, and early versus late RRT in patients with SA-AKI were evaluated. They defined early/late CRRT groups based on the time from admission to the start of RRT, i.e., those in whom RRT was initiated within or after 24 hours of SA-AKI confirmation. Renal recovery at 90 days in the early group was higher than in the late group (87% vs. 38.5%, p=0.032). When they compared early and late groups based on the KDIGO stage in which RRT was instituted, i.e., early (KDIGO stage II, n=13) and late KDIGO stage III (n=25), the authors found that both groups showed comparable outcomes, like renal recovery (p=0.153) and mortality (p=0.89) at 90 days [22].

Thus, we identified only two studies that examined the roles of early versus late CRRT in patients with SA-AKI, both of which were retrospective [21,22]. Of these, only one was labeled as early or late according to KDIGO guidelines for AKI [21]. Unlike us, the authors found that all outcome parameters studied, including mortality and renal recovery, were comparable. Being a retrospective study, it suffers from the usual pitfalls, such as recall bias, difficulty controlling confounders, and limitations in data quality due to data collected not being designed for future research questions.

Early institution of CRRT may be beneficial, as it would prevent fluid overload, eliminate toxins, establish acid-base homeostasis, attenuate systemic inflammation, and prevent other complications of AKI. Further, it is claimed that there is a possibility of washout of inflammatory mediators from the plasma due to early CRRT, as these are eliminated through CRRT and could also protect the kidneys and other organs [8,20].

We used regional citrate anticoagulation for CRRT in all patients. Anticoagulation with heparin is fraught with the risk of bleeding to the tune of 30-50% and resulting mortality of 15%. RCA offers a safer alternative because of prolonged filter life and fewer bleeding complications [23,24].

Limitations of our study

Our study was limited to one center. The study was underpowered to detect modest differences in mortality. Exclusion of very early deaths and missing cases may result in a bias towards a survival benefit in one group. Also, the study has a short follow-up (28 days), lacks long-term renal outcomes, and has limited adjustment for confounding factors. So, it cannot be said with certainty whether early CRRT confers a mortality benefit or delays mortality.

## Conclusions

From the analysis of data collected in our study, we may conclude that in this single-center prospective cohort of patients with S-AKI, early initiation of CRRT (KDIGO I/II) was associated with lower 28-day mortality compared to later initiation (KDIGO III). Given the small sample size and observational design, these findings should be considered hypothesis-generating and warrant confirmation in larger multicenter trials.
